# HGF, IL-1α, and IL-27 Are Robust Biomarkers in Early Severity Stratification of COVID-19 Patients

**DOI:** 10.3390/jcm10092017

**Published:** 2021-05-08

**Authors:** Álvaro Tamayo-Velasco, Pedro Martínez-Paz, María Jesús Peñarrubia-Ponce, Ignacio de la Fuente, Sonia Pérez-González, Itziar Fernández, Carlos Dueñas, Esther Gómez-Sánchez, Mario Lorenzo-López, Estefanía Gómez-Pesquera, María Heredia-Rodríguez, Irene Carnicero-Frutos, María Fe Muñoz-Moreno, David Bernardo, Francisco Javier Álvarez, Eduardo Tamayo, Hugo Gonzalo-Benito

**Affiliations:** 1 Department of Hematology, University Clinical Hospital, 47003 Valladolid, Spain; alvarotv1993@gmail.com (Á.T.-V.); mpenarrubia@saludcastillayleon.es (M.J.P.-P.); ifuentegr@saludcastillayleon.es (I.d.l.F.); sperezgon@saludcastillayleon.es (S.P.-G.); 2 Department of Surgery, Faculty of Medicine, University of Valladolid, 47005 Valladolid, Spain; pedrojose.martinez@uva.es (P.M.-P.); mlorenzol@saludcastillayleon.es (M.L.-L.); egomezp@saludcastillayleon.es (E.G.-P.); maria_her_05@hotmail.com (M.H.-R.); tamayo@med.uva.es (E.T.); 3 BioCritic (Group for Biomedical Research in Critical Care Medicine), University of Valladolid, 47005 Valladolid, Spain; alvarez@med.uva.es (F.J.Á.); hgonzalob@saludcastillayleon.es (H.G.-B.); 4 IOBA (Institute of Applied Ophthalmobiology), University of Valladolid, 47011 Valladolid, Spain; itziar.fernandez@uva.es; 5 Department of Internal Medicine, University Clinical Hospital, 47003 Valladolid, Spain; jduenas@saludcastillayleon.es; 6 Department of Anaesthesiology & Critical Care, University Clinical Hospital, 47003 Valladolid, Spain; 7 Department of Anaesthesiology & Critical Care, University Hospital, 37007 Salamanca, Spain; 8 Research Unit, University Clinical Hospital, 47003 Valladolid, Spain; icarnicerof@saludcastillayleon.es (I.C.-F.); mfmunozm@saludcastillayleon.es (M.F.M.-M.); 9 Institute of Health Sciences of Castile and Leon (IECSCYL), 47003 Valladolid, Spain; 10 Mucosal Immunology Laboratory, Institute of Biology and Molecular Genetics (IBGM), University of Valladolid, 47005 Valladolid, Spain; d.bernardo.ordiz@gmail.com; 11 Pharmacological Big Data Laboratory, Pharmacology, Faculty of Medicine, University of Valladolid, 47005 Valladolid, Spain

**Keywords:** coronavirus disease 2019, cytokines, severity, prognosis, mortality

## Abstract

Pneumonia is the leading cause of hospital admission and mortality in coronavirus disease 2019 (COVID-19). We aimed to identify the cytokines responsible for lung damage and mortality. We prospectively recruited 108 COVID-19 patients between March and April 2020 and divided them into four groups according to the severity of respiratory symptoms. Twenty-eight healthy volunteers were used for normalization of the results. Multiple cytokines showed statistically significant differences between mild and critical patients. High HGF levels were associated with the critical group (OR = 3.51; *p* < 0.001; 95%CI = 1.95–6.33). Moreover, high IL-1α (OR = 1.36; *p* = 0.01; 95%CI = 1.07–1.73) and low IL-27 (OR = 0.58; *p* < 0.005; 95%CI = 0.39–0.85) greatly increased the risk of ending up in the severe group. This model was especially sensitive in order to predict critical status (AUC = 0.794; specificity = 69.74%; sensitivity = 81.25%). Furthermore, high levels of HGF and IL-1α showed significant results in the survival analysis (*p* = 0.033 and *p* = 0.011, respectively). HGF, IL-1α, and IL 27 at hospital admission were strongly associated with severe/critical COVID-19 patients and therefore are excellent predictors of bad prognosis. HGF and IL-1α were also mortality biomarkers.

## 1. Introduction

In December 2019, a new strain of coronavirus, severe acute respiratory syndrome coronavirus 2 (SARS-CoV-2), was recognized to have emerged in Wuhan, China. Along with SARS-CoV [1] and Middle East respiratory syndrome-coronavirus (MERS-CoV), SARS-CoV-2 is the third coronavirus that causes severe respiratory disease in humans, called coronavirus disease 2019 (COVID-19) [2]. The epidemiology of the disease is not completely understood [3]. After a median incubation period of approximately 5 days, around half of patients present mild or no symptoms [4]. The others present moderate or severe respiratory disease including 20% of them who present serious illness with high fever and pneumonia [5], leading to acute respiratory distress syndrome (ARDS) [6].

Although its pathophysiology has not been fully understood [7], it is clear now that COVID-19 pathology arises from a primary deficit in type I interferon production followed by a dysregulated monocyte/macrophage infiltration which, in turn, drive an exacerbated adaptive immune response [8].Viral infection leads to rapid activation of innate immune cells, especially in patients who develop severe disease. The infection induces lymphocytopenia that primarily affects CD4^+^ T cells, including effector, memory, and regulatory T cells 3 [9,10]. Some biomarkers are related to moderate and severe COVID-19 infection like low lymphocytes absolute numbers [11] or increased levels of serum C-reactive protein (CRP), hypoalbuminemia, alanine aminotransferase, lactate dehydrogenase, ferritin, and/or D-dimer [12,13]. Indeed, these patients display increased levels of proinflammatory cytokines in serum like IL-1B, IL-6, IL-12, IFNγ, IP10, or MCP1/CCL2 [14,15] which are related to T helper 1 (Th1) cell responses. Moreover, the more severe patients (including those which require ICU admission) display higher plasma levels of GCSF, IP10, MCP1, MIP1A, and TNFα suggesting an association with the severity degree [16,17,18].

Based on this background, studies attribute the systemic impact of COVID-19 disease to a cytokine storm; a kind of ARDS induced by cytokine release syndrome (SRC) [19] or hemophagocytic lymphohistiocytosis (SHLH) [20], similar to that described in SARS-CoV and MERS-CoV patients. In this regard, and in order to confirm this point, most studies focused on the characterization of the cytokine response in COVID-19 patients are retrospective, present small series of patients, and/or are focused on a limited number of cytokines making them not suitable to understand the pathogenesis characterizing the cytokine release syndrome [5,12,16,18]. Moreover, the identification of prognosis biomarkers remains an urgent need.

In this regard, here we aimed to perform a cytokine array in plasma samples from a prospective COVID-19 cohort, aiming not just to characterize the cytokine storm but also to identify the early biomarkers of severity as well and mortality outcome.

## 2. Material and Methods

### 2.1. Patient Selection

A total of 108 adult patients, over 18 years, who were diagnosed with COVID-19 and admitted at the “Hospital Clínico Universitario” (Valladolid, Spain) were prospectively recruited between 24th of March and 11th of April 2020. Positive result in severe acute respiratory syndrome coronavirus 2 (SARS-CoV-2) infection was confirmed in all patients by polymerase chain reaction on nasopharyngeal samples. Patients with of other acute diseases, infections, or chronic terminal illness were not included. In addition, we also included 28 age- and gender-matched healthy volunteers for the normalization of the analytical data of the cytokines. The study was approved by the Hospital’s Clinical Ethics Committee (CEIm) and the approval was obtained from all study participants (cod: PI 20-1717). This study followed the code of ethics of the World Medical Association (Declaration of Helsinki).

### 2.2. Biological Samples

We prospectively recruited plasma samples from each patient at 9 am immediately after their first night in the hospital in order to prevent circadian variations. Blood was collected in 3.2% sodium citrate tubes and centrifuged at 2000× *g* for 20 min at room temperature. The resulting plasma was aliquoted and directly frozen at −80 °C until used.

### 2.3. Degrees of Severity

Patients were divided into four groups based on their subsequent clinical outcome according to the severity of the respiratory symptoms: (i) Mild (*n* = 34): pneumonia—Adolescent or adult with clinical signs of pneumonia (fever, cough, dyspnea, fast breathing) but no signs of severe pneumonia, including SpO2 ≥ 90% on room air; (ii) moderate (*n* = 26): adolescent or adult with clinical signs of pneumonia (fever, cough, dyspnea, fast breathing) plus one of the following: respiratory rate > 30 breaths/min; severe respiratory distress; or SpO2 < 90% on room air- [200 mmHg < PaO2/FiO2a ≤ 300 mmHg (with PEEP or CPAP ≥ 5 cmH2O, or non-ventilated]; (iii) severe (*n* = 16): adolescent or adult with clinical signs of pneumonia (fever, cough, dyspnea, fast breathing) plus one of the following: respiratory rate > 30 breaths/min; severe respiratory distress; or SpO2 < 90% on room air [100 mmHg < PaO2/FiO2 ≤ 200 mmHg (with PEEP ≥ 5 cmH2O, or non-ventilated]; (iv) critical (*n* = 32): adolescent or adult with clinical signs of pneumonia (fever, cough, dyspnea, fast breathing) plus one of the following: respiratory rate >30 breaths/min; severe respiratory distress; or SpO2 < 90% on room air [100 mmHg < PaO2/FiO2 ≤ 200 mmHg (with PEEP ≥ 5 cmH2O] and mechanical ventilation. This classification is based on the WHO guide [21].

### 2.4. Cytokines and Chemokines Analysis

Plasma aliquots at hospital admission were analyzed, in duplicate, for the quantification of soluble mediators by the kit 45-plex Human XL Cytokine Luminex Performance Panel (R&D) following the manufacturer’s guidelines and recommendations. Cytokines or chemokines included in the Panel were BDNF, EGF, Eotaxin (also known as CCL11), FGF-2, GM-CSF, GRO-α (CXCL1), HGF, IFN-α, IFN-γ, IL-1α, IL-1β, IL-10, IL-12 p70, IL-13, IL-15, IL-17a (CTLA-18), IL-18, IL-1RA, IL-2, IL-21, IL-22, IL-23, IL-27, IL-31, IL-4, IL-5, IL-6, IL-7, IL-8 (CXCL8), IL-9, IP-1 beta (CCL4), IP-10 (CXCL10), LIF, MCP-1 (CCL2), MIP-1α (CCL3), NGF-β, PDGF-BB, PIGF-1, RANTES (CCL5), SCF, SDF-1α, TNF-α, TNF-β, VEGF-A, VEGF-D.

### 2.5. Variables

Demographic, clinical and analytical data (leukocytes, lymphocytes, neutrophils, platelets, bilirubin, creatinine, glucose, troponin Ths, C-reactive protein (CRP), lactate dehydrogenase (LDH), ferritin, procalcitonin, and D-dimer) of each patient were also recorded to describe the clinical phenotype.

### 2.6. Statistical Analysis

Statistical analysis was performed by a PhD-licensed statistician (co-author IF) using the R statistical package version 4.0.2 (R Core Team; Foundation for Statistical Computing, Vienna, Austria; URL: https://www.R-project.org/, accessed on 5 April 2021). Statistical significance was set at *p* ≤ 0.05.

To impute cytokine values below the assay detection limit, robust regression on order statistics was used: this method performs a regression to impute low values assuming log-normal quantiles for samples with a detection rate of at least 20%, after checking that the data follow a log-normal distribution. To accomplish this, the non-detects and data analysis (NADA) R package was used [Lopaka, 2017] [22]. Molecules detected in less than 20% of the samples were not statistically analyzed any further. Cytokine expression data were transformed using the logarithmic base 2 scale. Continuous variables are represented as [median, (interquartile range, IQR)], while categorical variables are represented as [%, (*n*)].

The strength of each biomarkers was evaluated at the individual level to determine the pulmonary severity of the patient. The main variable was severity, which is an ordinal variable with four levels. The first model to be fitted was an ordinal logistic regression model or proportional odds model [Hosmer & Lemeshow, 2000]. To confirm this, the proportional odds model was compared with a multinomial logistic regression one through the likelihood ratio test. However, in none of the cases was it possible to assume this hypothesis, so multinomial models were fitted.

Biomarkers associated with the severity at the 10% significance level were identified as potential biomarkers and they were evaluated simultaneously to fit a multivariable model.

The leave-one-out-cross-validation (LOOCV) procedure was used to estimate the prediction accuracy of the final fitted models, and receiver operation characteristic (ROC) curve analysis was used to assess their discriminate ability. The final models were evaluated according to the area under the ROC curve (AUC). In addition, sensitivity and specificity were obtained by setting an optimal threshold.

A survival analysis was also performed with the final panel of cytokines identified by the multivariable models. The outcome was tested related to T2 (time since the hospitalization until death/end of the survey). For survivals, the days of follow-up were hospitalization time or 28 days in outpatients after leaving the hospital. The Kaplan-Meier survival function was used by the log-rank test to determine differences in survival rates, considered different when *p* < 0.05. The cut-off point is established in each cytokine selecting the one with the greatest area under the ROC curve (AUC) in the individual model.

## 3. Results

Our cohort had a median age of 67 years, mostly male (63.26%). The control group of healthy volunteers had a median age of 61 years and most of them (57.1%) were also male. Patients were divided into four severity degrees based on the subsequent outcome during their hospital stay: (i) mild [*n* = 34, 31.5%, IC 95% (23.07–41.23)], (ii) moderate [*n* = 26, 24.1%, IC 95% (16.59–33.43)], (iii) severe [*n* = 16, 14.8%, IC 95% (8.96–23.24)], and (iv) critical [*n* = 32, 29.6%, IC 95% (21.43–39.3)] defined by their need of oxygen supplementation.

Patient clinical and analytical profile at hospital admission are shown in Table 1. Patient’s group did not differ regarding age, gender, or comorbidities. However, ferritin, D-dimer, leukocytes, neutrophils, procalcitonin, and glycaemia displayed higher levels with the greater severity. On the other hand, lymphocytes, platelets, and PaO2/FiO2 were decreased in critical patients. Length of hospital stay was also increased according to the severity (8 days, 8 days, 13.5 days and 26.5 days respectively). Mortality was also higher in severe [50% (8 patients)] and critical [43.8% (14 patients)] patients compared with moderate [3.8% (1 patients)] and mild [2.9% (1 patients)].

**Figure 1:**
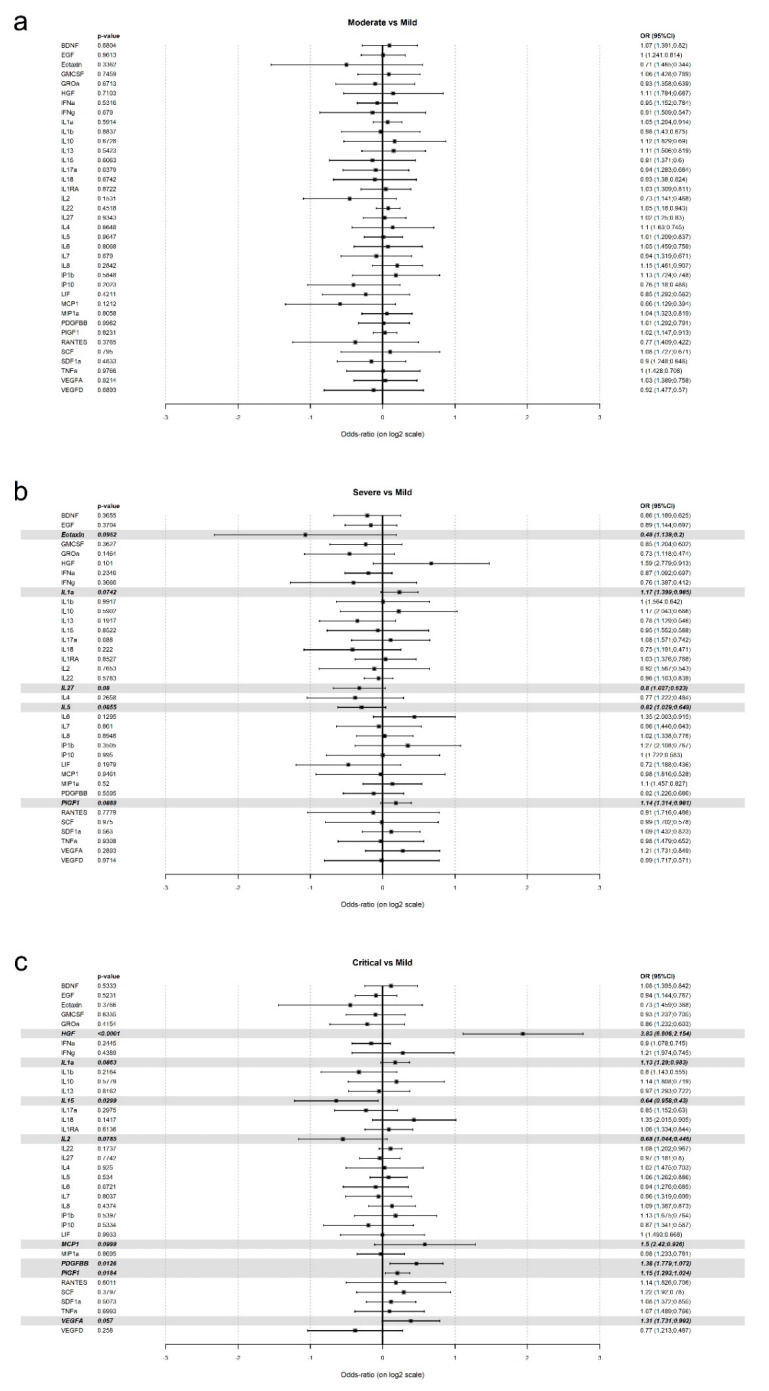
Individual multinomial models using the mild group as a reference. (**a**) Moderate. (**b**) Severe. (**c**) Critical.

To impute low values assuming log-normal quantiles for samples, a detection rate of at least 20% is required. Under these conditions, eight cytokines (FGF-2, IL-12, IL-21, IL-23, IL-31, IL-9, NGF-β, and TNF-β) were therefore excluded from the analysis (Supplement Table S1). Median values of each cytokine according to the severity degree are shown in Supplement Table S2. Based on a likelihood ratio test (Supplement Table S3), the most plausible model in all cases is the multinomial one. Hence, we performed individual multinomial models using the mild group as a reference (Figure 1a–c).

The comparison of mild with moderate (Figure 1a) or severe (Figure 1b) patients was not statistically significant for any of the studied cytokines although Eotaxin, IL1-α, Il-27, IL-5, and PIGF1 were borderline in the latter. Nevertheless, the comparison of mild with patients who ended up critical displayed statistical differences for several cytokines. Hence, HGF, PDGFBB, PIGF1, IL-1α, MCP1, and VEGFA were over-expressed at hospital admission in the critical group by 3.83, 1.38, 1.15, 1.13, 1.5, 1.31 times respectively. On the contrary, IL-15 and IL-2 were under-expressed in the critical patients at hospital admission by 1.56 (1/0.64) and 1.47 (1/0.68).

The best multivariable model based on these molecules is the one with four cytokines: HGF, IL1a, IL2, and IL27 (Table 2). The sex- and age-adjusted odds ratios are shown in Table 3. This analysis revealed an association between high levels of HGF and IL-1α coupled with low levels of IL-27 at hospital admission as bad prognosis predictors as these patients ended up in the severe or the critical group. In this regard, patients with twice the expression of HGF at admission had 3.51 times more chances of being critical than mild [OR: 3.51; *p* < 0.001; CI 95% (1.95–6.33)]. In a similar manner, if IL-1α [OR: 1.36; *p* = 0.01; CI 95% (1.07–1.73)] or IL-27 [OR: 0.58; *p* < 0.005; CI 95% (0.39–0.85)] were over- or under-expressed at admission, the risk of being in the severe group was 1.36 and 1.74 respectively (1/0.5753) referred to as the mild group.

**Figure 2:**
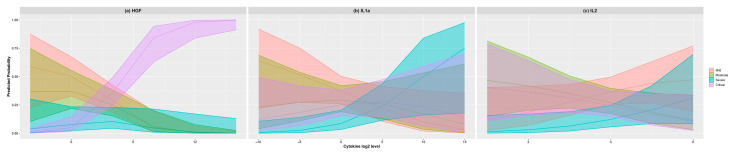
Effect plots of the estimated probabilities of belonging to each severity group according to the level of HGF (**a**), IL-1α (**b**), and IL-27 (**c**). The log2 level of each cytokine is measured in pg/mL.

The fitted models are used to estimate the predicted probabilities and their associated confidence bands of severity group. These estimated probabilities are visualized as effect plots in Figure 2a–c. We clearly see how the chances of ending up in a critical condition were directly related to higher HGF levels at admission. Hence, HGF levels above 128 pg/mL (2^7^) imply a 25% chance of being critical while levels above 223 pg/mL increase that critical risk up to 50%. On the contrary, patients with HGF levels below 64 pg/mL (2^6^) have no risk (practically 0%) of ending up critical. In the same manner, low IL-1α levels at admission had a probability over 37% of being mild, while IL-1alpha levels over 1024 pg/mL (2^10^) had 50% chances of being in the severe group. Last, but not least, lower levels of IL-27 at admission were also associated with the severe group since level under 1 are reflected in a 50% chance of belonging to the severe group while IL-27 levels over 64 pg/mL (2^6^) decrease that risk to practically 0%.

**Figure 3:**
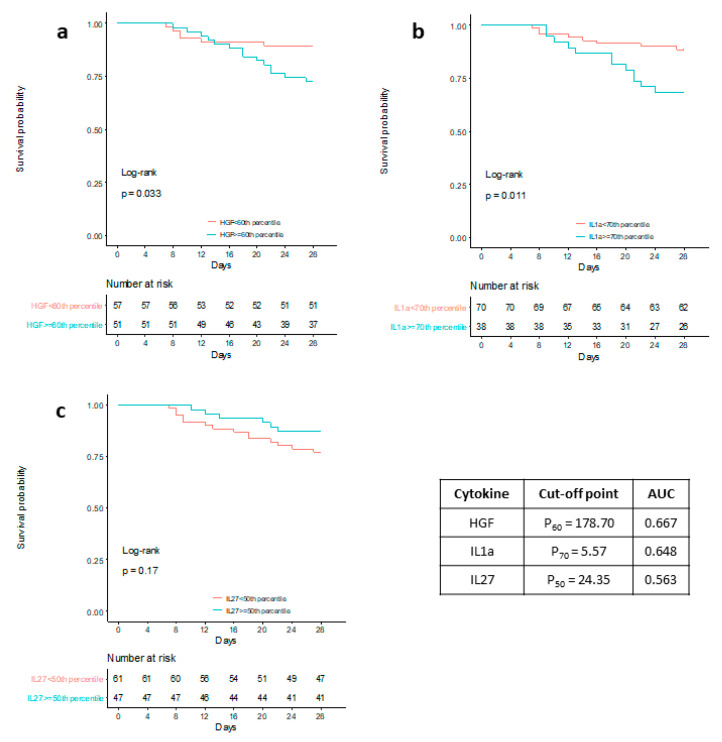
Kaplan-Meier survival curves for HGF (**a**), IL-1α (**b**), IL-27 (**c**).

Internal validation by the LOOCV procedure shows that AUC is significantly greater than 0.5 in all severity groups (Table 4), especially in severe group (AUC 0.730) and critical group (0.794). This model is especially sensitive in order to classify patients who end up critical (sensitivity = 81.25%). Last, but not least, the survival analysis taking into account the three statistically significance cytokines included in the multivariable model was significant for HGF and IL-1α (Figure 3a,b) but not IL-27 (Figure 3c).

## 4. Discussion

Here we have described, after performing a 45-plex cytokine array on plasma samples from 108 patients at hospital admission, that five cytokines are statistically significantly different according to the degrees of severity in COVID-19. Indeed, high levels of HGF and IL-1α coupled with low levels of IL-27 at admission can predict bad clinical outcome referred to the patient subset with better prognosis, being especially important the high level of HGF as predictors of admission in intensive care units. Moreover, this multivariate model was especially sensitive in order to identify those patients who end up in a critical status (AUC = 0.794; specificity 69.74%; sensitivity = 81.25%) following hospital admission. Last, but not the least, we have also described how the combination of high levels of HGF IL-1 α at admission can predict mortality, showing significant results in the survival analysis (*p* = 0.033 and *p* = 0.011 respectively).

During the last months, several studies have tried to understand the cytokine profile in patients with COVID-19. Most of them relate severity of lung disease to high levels of multiple cytokines in blood, according to what has been defined as a cytokine storm. Indeed, even some authors describe three different clinical phenotypes of COVID-19 based on cytokines levels [23]. In this regard, Huang et al. suggest that the cytokine storm is associated with severity after analyzing 27 cytokines in 41 patients as ICU patients had higher plasma levels of IL-2, IL-7, IL-10, GSCF, IP10, MCP1, MIP1A, and TNFα [18]. In a similar manner, Liu et al. studied 40 patients, 13 of them severe, and found increased plasma levels of IL-6, IL-10, IL-2, and IFN-γ levels in severe compared to mild cases [24]. Zhao et al. included 71 patients, (53 mild and 18 severe) referred 18 healthy volunteers describing that IL-1RA and IL-10 correlated with disease severity, while Zhang et al. analyzed in 326 patients finding higher levels of IL-6 and IL-8 in severe or critical patients [25]. Nevertheless, these studies display several limitations like small sample sizes, the study of few numbers of cytokines, and the lack of well-defined severity degrees. Moreover, patients who required mechanical ventilation were not usually differentiated from patients with severe disease despite this aggressive intervention increases cytokine levels. Last, but not least, these studies usually applied basic statistical approaches. Therefore, and in order to overcome these limitations, we hereby have analyzed in duplicate the plasma levels of 45 cytokines from an extremely well-categorized cohort of 108 COVID-19 patients which were classified into severity groups based on their clinical evolution defined by objective criteria, at the time that we also performed an exhaustive statistical analysis. Hence, we have considered all confounders by using both univariate and multivariate regression analysis showing, at least, an internal validation.

Other studies have performed a similar approach to the one here described, like the one by Han et al. that classified 102 patients into moderate, severe, and critical groups according to their symptoms. It also presented a control group of healthy volunteers. Such study showed higher serum levels of TNF-α, IFN-γ, IL-2, IL-4, IL-6, IL-10, and CRP referred to controls. Using a logistic regression analysis, IL-6 and IL-10 were found to predict disease severity and the internal validation could further confirm this result [26]. However, they only analyzed six cytokines and a duplicate analysis was not performed on each sample. In a similar manner, Meizlish et al. analyzed a cohort with 49 adult patients (40 in the medical intensive care unit (ICU) and 9 in non-ICU units), as well as 13 non-COVID-19 healthy volunteers. They analyzed 78 circulating proteins with immunologic functions. Their study identified a neutrophil activation signature composed of neutrophil activators (G-CSF, IL-8) and effectors (resistin (RETN), lipocalin-2 (LCN2) and hepatocyte growth factor (HGF)), which had the greater power to identify critically ill patients [27]. As default, the small number of patients and the different degrees of pulmonary severity do not differ in non-ICU patients.

Based on the results displayed by these two studies, and in agreement with ours, we can conclude that there is no specific cytokine pattern correlating with the disease severity. On the one hand, high levels of HGF were associated with a risk of up to 3.5 times of being critical with mechanical ventilation. This growth factor, that has already been related to severity in other studies, primarily elicits its effects on epithelial cells. In a similar manner, IL-1α, which is a pro-inflammatory cytokine from the innate immune system mainly produced by macrophages but also epithelial cells, can also predict a bad prognosis and disease outcome. Hence, both cytokines could be reflecting the tissue damage elicited by the macrophage infiltration to the lungs [28,29]. Indeed, these findings suggest the implication of non-immune cells in COVID-19 in agreement with the results from Lucas et al. [30] who proved how increased stromal growth factors involved in tissue repairing were associated with a favorable immune signature. Hence, it seems obvious now that the crosstalk between immune and stromal cells in the lungs may shape the fate of the immune response and, with that, the outcome of the patient evolution.

We have also found how low level of IL-27, which belongs to the IL-12 family and is therefore involved in Th1 differentiation, is a good prognosis biomarker in COVID-19 patients. Together, these results suggest that, although in our hands the cytokine storm may not be the trigger of the bilateral pneumonia, there is certainly a mixed and altered cytokine profile which drives disease progression and inflammation as highlighted by the fact that high HGF levels combined with low IL-27 levels are revealed as early mortality markers. We are nevertheless aware that we have not found increased levels of IL-6 levels to be relevant in our cohort as many studies have already reported [31,32]. One possible explanation is that, in our case, we simultaneously determined the levels of 45 cytokines in a large cohort and performed a multivariate analysis. Hence, the single effect of IL-6 may be diluted in favor of the combined of several other cytokines. Nevertheless, the moment when the samples were obtained may also provide an explanation. Indeed, our cohort was recruited during the worst days of the pandemics in Spain between March and April 2020, when some patients were immediately transferred into the ICU after arriving to the hospital. Hence, given that our cohort also displayed high levels of CRP (a downstream mediator of IL-6), we cannot discard the possibility that IL-6 was higher and driving inflammation in previous stages of the disease before the patients were admitted to the hospital and therefore recruited.

Since the beginning of this health crisis, treatment strategies in the most severe cases were aimed at blocking interleukins like IL-6 (Tocilizumab), IL-1 (Anakinra), and TNFα (Infliximab, Adalimumab, etc.,) [33]. The REMAP-CAP and RECOVERY studies show modest but significant improvement in mortality [34,35] and these findings were confirmed in the Cochrane review showing high certainty of improvement in 28 day mortality in patients who received IL-6 blockade (RR 0.89, 95% CI 0.82–0.97). The use of dexamethasone at two drops for 10 days decreased mortality at day 28 in patients who were receiving invasive mechanical ventilation [36]. Nevertheless, and as a corticosteroid, this approach did not identify the key immune components involved in this process. According to this, and the results hereby reported, it is to be expected that these strategies entail a modest reduction in mortality since increased levels of IL-6, IL-1, or TNFα are not directly responsible to drive disease severity in these patients. Therefore, and although the increased levels of plasma cytokines in COVID-19 patients has been largely reported, the identification of disease progression and severity biomarkers remains an urgent need. In this regard, we hereby report that HGF, IL1α, and IL27 contribute to the deterioration of the disease and the adverse outcome of COVID-19 revealing these three compounds as novel biomarkers but as future therapeutic targets in COVID-19.

We are aware of the main limitations of our study. (i) Our study did not include a large sample size. Perhaps, we should have performed previously a statistical power analysis. Nevertheless, our sample size is consistent with previous reports [18,24,25,30]. We were very careful with the recruitment and analysis of plasma samples, at the same time each day and with a duplicate analysis, in order to avoid circadian variations. Therefore, we intended to get samples as homogeneous as possible. (ii) Lack of external validation. Therefore, we consider that validating the model in a different cohort of patients in the future would be essential to give consistency to the results. (iii) The most relevant buffering system in the COVID-cytokine storm is the IL-6: sIL-6R:sgp130 system in trans signaling, which has been described in recent publications [37,38]. Thus, an inherent limitation of these multi-PLEX cytokine studies is that they typically only measure the cytokine itself, whereas there are other aspects of these cytokine signaling pathways that are omitted.

Our study characterized the plasma cytokine profile of COVID-19 patients at hospital admission, based on their subsequent clinical evolution into four well-defined degrees of severity, revealing that HGF, IL-1α, and IL27 were strongly associated with disease severity and could be used as excellent predictors of bad prognosis. Indeed, HGF and IL-1α are also mortality biomarkers. Therefore, the early detection of HGF, IL-1α, and IL27 plasma levels in patients in COVID-19 patients can provide useful information for getting quickly intensive treatment as well as providing possible therapeutic targets.

## Figures and Tables

**Table 1:** Clinical characteristics of the patients.

	Mild (*n* = 34)	Moderate (*n* = 26)	Severe (*n* = 16)	Critical (*n* = 32)	*p* Value
Age [median (IQR)]	68 (18)	65 (17)	75 (14)	70 (16)	0.121
Male [%(n)]	45.2% (14)	61.5% (16)	62.5% (10)	54.8% (17)	0.568
**-Comorbidities, [%(*n*)]**					
Use of tobacco	8.80% (3)	3.80% (1)	6.3% (1)	12.5% (4)	0.679
Use of alcohol	5.90% (2)	0% (0)	0% (0)	3.1% (1)	0.488
Coronary cardiopathy	8.8% (3)	11.5% (3)	12.5% (2)	6.30% (2)	0.870
Valvular disease	5.90% (2)	0% (0)	12.5% (2)	0% (0)	0.104
Atrial fibrillation	17.6% (6)	3.80% (1)	18.8% (3)	6.3% (2)	0.206
Diabetes	11.8% (4)	11.5% (3)	18.8% (3)	25% (8)	0.435
Hypertension	50% (17)	34.6% (9)	56.3% (9)	46.9% (15)	0.521
Liver disease	0% (0)	0% (0)	0% (0)	6.3% (2)	-
COPD	0% (0)	7.7% (2)	18.8% (3)	6.3% (2)	0.094
Kidney disease	2.90% (1)	0% (0)	0% (0)	6.3% (2)	0.452
Asthma	11.8% (4)	3.80% (1)	0% (0)	3.1% (1)	0.268
**-Laboratory, [median (IQR)]**					
Glucemia (mg/dL)	90 (13)	109 (56)	120 (59)	209 (99)	<0.001
Leukocytes (n º/mL)	4620 (2880)	6990 (3020)	6630 (3480)	7900 (8680)	<0.001
Lymphocytes (n º/mL)	1000 (430)	1000 (1000)	1120 (531)	440 (455)	<0.001
Neutrophil (n º/mL)	3215 (2420)	4945 (2380)	5315 (3450)	7045 (7800)	<0.001
Procalcitonin (ng/mL)	0.06 (0)	0.05 (0)	0.15 (1)	0.24 (0)	<0.001
CRP (mg/L)	76.5 (88)	73.5 (106)	127.0 (113)	97.0 (153)	0.250
Creatinine (mg/dL)	0.81 (0)	0.78 (0)	0.88 (0)	0.89 (1)	0.242
Total bilirubin (mg/dL)	0.40 (0)	0.5 (0)	0.65 (0)	0.50 (1)	0.187
Platelet (cell/mm^3^)	(82,000)	232,500 (171,000)	198,500 (108,500)	216,500 (108,000)	0.005
Ferritin (ng/mL)	587 (600)	674 (906)	1025 (938)	1700 (1093)	<0.001
D-dimer (ng/mL)	547 (333)	693 (702)	1083 (1398)	1847 (1823)	<0.001
PaO2/FiO2	371 (48)	304 (94)	238 (102)	127 (44)	<0.001
**-Hospital meters, [median (IQR)]**					
Length of hospital stay (days)	8 (4)	8 (6)	13.5 (10)	26.5 (39)	<0.001
Length of ICU stay (days)	0 (0)	0 (0)	0 (0)	18.5 (14)	0.172
Intubation time (days)	0 (0)	0 (0)	0 (0)	14 (12)	0.172
**-Mortality, [%(*n*)]**					
90-days mortality	2.9% (1)	3.8% (1)	50% (8)	43.8% (14)	<0.001
28-days mortality	0% (0)	3.8% (1)	43.8% (7)	37.5% (12)	<0.001

Continuous variables are represented as [median, (interquartile range, IQR)]; categorical variables are represented as [%, (*n*)]; COPD, chronic obstructive pulmonary disease; CRP, C-reactive protein.

**Table 2:** Identification of the best multivariable model following AIC (“Akaike’s Information Criterion”).

	Int.	Age	Sex	HGF	IL-1α	IL-15	IL-2	IL-27	IL-5	MCP1	PDGFBB	PIGF1	VEGFA	AIC
M0	√	√	√											301.7077
M1	√	√	√	√										268.1021
M2	√	√	√	√					√					268.3859
M3	√	√	√	√		√			√					265.8642
M4	√	√	√	√	√		√	√						264.8347
M5	√	√	√	√		√		√			√	√		265.6192
M6	√	√	√	√		√	√	√			√	√		267.9954
M7	√	√	√	√		√	√	√		√	√	√		271.669
M8	√	√	√	√	√	√	√	√			√	√	√	274.8803
M9	√	√	√	√	√	√	√	√		√	√	√	√	278.3977
M10	√	√	√	√	√	√	√	√	√	√	√	√	√	282.1787

Int, intercept.

**Table 3:** Different multivariable models according to the degrees of severity.

Severity	Effect	*p* Value	OR	CI 95%	
				Low	High
Moderate	Age	0.573	0.9883	0.9486	1.0296
	Sex = Female	0.1648	0.4618	0.1553	1.3735
	HGF	0.7528	1.0853	0.652	1.8066
	IL1a	0.4346	1.081	0.8891	1.3144
	*IL2*	*0.067*	*0.57*	*0.3124*	*1.0401*
	IL27	0.487	1.1148	0.8206	1.5144
Severe	Age	0.0452	1.0687	1.0014	1.1405
	Sex = Female	0.1504	0.3517	0.0847	1.4611
	HGF	0.2144	1.5301	0.7818	2.9946
	*IL1a*	*0.0109*	1.3634	*1.0741*	*1.7308*
	IL2	0.4125	1.4144	0.6172	3.2414
	*IL27*	*0.0057*	*0.5753*	*0.3888*	*0.8511*
Critical	Age	0.13	0.9615	0.9139	1.0116
	Sex = Female	0.758	0.8242	0.241	2.8192
	HGF	<0.0001	3.5122	1.9495	6.3276
	IL1a	0.1977	1.134	0.9365	1.3731
	IL2	0.1105	0.5776	0.2943	1.1334
	IL27	0.8571	0.9677	0.6772	1.383

CI, confidence interval; OR, odds ratio.

**Table 4:** Internal validation in each degree of severity using the AUC (area under the ROC curve).

	Mild Threshold: 0.3597126			Moderate Threshold: 0.2513263			Severe Threshold: 0.1438022			Critical Threshold: 0.2084408		
	Value	CI 95%		Value	CI 95%		Value	CI 95%		Value	CI 95%	
		Lower	Higher		Lower	Higher		Lower	Higher		Lower	Higher
AUC	0.647	0.535	0.759	0.602	0.477	0.727	0.730	0.624	0.837	0.794	0.701	0.888
Sensitivity (%)	58.82	42.28	75.37	53.85	34.68	73.01	62.5	38.78	86.22	81.25	67.73	94.77
Specificity (%)	70.27	59.86	80.68	65.85	55.59	76.12	73.91	64.94	82.89	69.74	59.41	80.07
Accuracy (%)	66.67	57.78	75.56	62.96	53.86	72.07	72.22	63.77	80.67	73.15	64.79	81.51

CI, confident interval.

## Data Availability

The datasets generated during and/or analyzed during the current study are available from the corresponding author on reasonable request.

## Supplementary Materials

The following are available online at https://www.mdpi.com/article/10.3390/jcm10092017/s1, Table S1: Cytokine/chemokine detection percentage. Table S2: Comparison between the value of cytokines according to their degree of severity. Table S3: Likelihood-ratio test (LRT) to check the assumption of proportional odds by comparing the proportional odds model with a multinomial model.
