# Supplementary material for: HGF, IL-1α, and IL-27 Are Robust Biomarkers in Early Severity Stratification of COVID-19 Patients
Source: J Clin Med. 2021 May 8;10(9):2017. doi: 10.3390/jcm10092017 (PMC8125923; doi:10.3390/jcm10092017)
Supplement: Supplementary file 1 [file jcm-10-02017-s001.zip › jcm-1210729-supplementary.pdf]

SUPPLEMENTARY CONTENT

Table S1: Cytokine/Chemokine detection percentage (Those marked do not exceed 20% detection and are excluded from the analysis).

Cytokine - Chemokine	Group	n	%	CI 95%	
				Low	High
BDNF	Mild	33	97.1	82.95	99.85
	Moderate	25	96.2	78.42	99.8
	Severe	16	100	75.93	100
	Critical	32	100	86.66	100
	Control	24	85.7	66.44	95.32
	Total	130	95.6	90.23	98.19
EGF	Mild	16	47.1	30.16	64.6
	Moderate	9	34.6	17.94	55.64
	Severe	6	37.5	16.28	64.13
	Critical	11	34.4	19.17	53.23
	Control	2	7.1	1.25	24.96
	Total	44	32.4	24.73	40.99
Eotaxin	Mild	34	100	87.36	100
	Moderate	26	100	83.98	100
	Severe	16	100	75.93	100
	Critical	32	100	86.66	100
	Control	28	100	84.98	100
	Total	136	100	96.58	100
**FGF2**	Mild	3	8.8	2.31	24.81
	Moderate	6	23.1	9.75	44.08
	Severe	2	12.5	2.2	39.59
	Critical	1	3.1	0.16	18
	Control	0	0	0	15.02
	Total	12	8.8	4.85	15.23
GMCSF	Mild	19	55.9	38.09	72.38
	Moderate	15	57.7	37.19	76.03
	Severe	11	68.8	41.48	87.87
	Critical	15	46.9	29.51	64.97
	Control	0	0	0	15.02
	Total	60	44.1	35.7	52.87
GROa	Mild	14	41.2	25.13	59.17
	Moderate	12	46.2	27.14	66.25
	Severe	8	50	28	72
	Critical	9	28.1	14.4	46.98
	Control	0	0	0	15.02
	Total	43	31.6	24.07	40.23
HGF	Mild	34	100	87.36	100
	Moderate	26	100	83.98	100
	Severe	16	100	75.93	100

**Cytokine - Chemokine**	Group	n	%	CI 95%	
				Low	High
	Critical	32	100	86.66	100
	Control	28	100	84.98	100
	Total	136	100	96.58	100
IFNa	Mild	20	58.8	40.83	74.87
	Moderate	11	42.3	23.97	62.81
	Severe	7	43.8	20.75	69.45
	Critical	11	34.4	19.17	53.23
	Control	1	3.6	0.19	20.24
	Total	50	36.8	28.79	45.5
IFNg	Mild	12	35.3	20.3	53.53
	Moderate	9	34.6	17.94	55.64
	Severe	6	37.5	16.28	64.13
	Critical	17	53.1	35.03	70.49
	Control	0	0	0	15.02
	Total	44	32.4	24.73	40.99
IL1a	Mild	23	67.6	49.37	82.02
	Moderate	18	69.2	48.1	84.91
	Severe	12	75	47.41	91.67
	Critical	26	81.2	62.96	92.14
	Control	7	25	11.43	45.22
	Total	86	63.2	54.5	71.21
IL1b	Mild	34	100	87.36	100
	Moderate	26	100	83.98	100
	Severe	16	100	75.93	100
	Critical	28	87.5	70.07	95.92
	Control	6	21.4	9.03	41.46
	Total	110	80.9	73.06	86.92
IL10	Mild	17	50	34.07	65.93
	Moderate	17	65.4	44.36	82.06
	Severe	11	68.8	41.48	87.87
	Critical	21	65.6	46.77	80.83
	Control	1	3.6	0.19	20.24
	Total	67	49.3	40.64	57.93
**IL12p70**	Mild	5	14.7	5.54	31.83
	Moderate	6	23.1	9.75	44.08
	Severe	4	25	8.33	52.59
	Critical	7	21.9	9.94	40.44
	Control	0	0	0	15.02
	Total	22	16.2	10.63	23.69
IL13	Mild	13	38.2	22.69	56.38
	Moderate	10	38.5	20.91	59.27
	Severe	6	37.5	16.28	64.13
	Critical	8	25	12.13	43.75
	Control	1	3.6	0.19	20.24

Cytokine - Chemokine	Group	n	%	CI 95%	
				Low	High
	Total	38	27.9	20.76	36.4
IL15	Mild	33	97.1	82.95	99.85
	Moderate	26	100	83.98	100
	Severe	16	100	75.93	100
	Critical	28	87.5	70.07	95.92
	Control	11	39.3	22.13	59.27
	Total	114	83.8	76.31	89.37
IL17a	Mild	33	97.1	82.95	99.85
	Moderate	26	100	83.98	100
	Severe	16	100	75.93	100
	Critical	30	93.8	77.78	98.91
	Control	7	25	11.43	45.22
	Total	112	82.4	74.68	88.15
IL18	Mild	32	94.1	78.94	98.97
	Moderate	25	96.2	78.42	99.8
	Severe	15	93.8	67.71	99.67
	Critical	31	96.9	82	99.84
	Control	17	60.7	40.73	77.87
	Total	120	88.2	81.32	92.92
IL1RA	Mild	32	94.1	78.94	98.97
	Moderate	24	92.3	73.4	98.66
	Severe	15	93.8	67.71	99.67
	Critical	30	93.8	77.78	98.91
	Control	4	14.3	4.68	33.56
	Total	105	77.2	69.07	83.77
IL2	Mild	31	91.2	75.19	97.69
	Moderate	23	88.5	68.72	96.97
	Severe	16	100	75.93	100
	Critical	23	71.9	53.02	85.6
	Control	5	17.9	6.77	37.58
	Total	98	72.1	63.6	79.24
**IL21**	Mild	7	20.6	9.34	38.41
	Moderate	7	26.9	12.35	48.05
	Severe	3	18.8	4.97	46.31
	Critical	5	15.6	5.9	33.55
	Control	3	10.7	2.81	29.37
	Total	25	18.4	12.46	26.13
IL22	Mild	6	17.6	7.39	35.17
	Moderate	6	23.1	9.75	44.08
	Severe	3	18.8	4.97	46.31
	Critical	12	37.5	21.66	56.25
	Control	4	14.3	4.68	33.56
	Total	31	22.8	16.23	30.93
**IL23**	Mild	4	11.8	3.84	28.39

Cytokine - Chemokine	Group	n	%	CI 95%	
				Low	High
	Moderate	2	7.7	1.34	26.6
	Severe	2	12.5	2.2	39.59
	Critical	1	3.1	0.16	18
	Control	0	0	0	15.02
	Total	9	6.6	3.26	12.55
**IL27**	Mild	13	38.2	22.69	56.38
	Moderate	8	30.8	15.09	51.9
	Severe	4	25	8.33	52.59
	Critical	8	25	12.13	43.75
	Control	1	3.6	0.19	20.24
	Total	34	25	18.15	33.29
**IL31**	Mild	3	8.8	2.31	24.81
	Moderate	3	11.5	3.03	31.28
	Severe	3	18.8	4.97	46.31
	Critical	0	0	0	13.34
	Control	0	0	0	15.02
	Total	9	6.6	3.26	12.55
IL4	Mild	9	26.5	13.51	44.65
	Moderate	9	34.6	17.94	55.64
	Severe	6	37.5	16.28	64.13
	Critical	7	21.9	9.94	40.44
	Control	0	0	0	15.02
	Total	31	22.8	16.23	30.93
IL5	Mild	16	47.1	30.16	64.6
	Moderate	12	46.2	27.14	66.25
	Severe	7	43.8	20.75	69.45
	Critical	14	43.8	26.84	62.12
	Control	1	3.6	0.19	20.24
	Total	50	36.8	28.79	45.5
IL6	Mild	27	79.4	61.59	90.66
	Moderate	20	76.9	55.92	90.25
	Severe	14	87.5	60.41	97.8
	Critical	19	59.4	40.79	75.78
	Control	2	7.1	1.25	24.96
	Total	82	60.3	51.53	68.47
IL7	Mild	32	94.1	78.94	98.97
	Moderate	22	84.6	64.27	94.95
	Severe	15	93.8	67.71	99.67
	Critical	31	96.9	82	99.84
	Control	4	14.3	4.68	33.56
	Total	104	76.5	68.28	83.13
IL8	Mild	11	32.4	17.98	50.63
	Moderate	9	34.6	17.94	55.64
	Severe	8	50	28	72

Cytokine - Chemokine	Group	n	%	CI 95%	
				Low	High
	Critical	11	34.4	19.17	53.23
	Control	3	10.7	2.81	29.37
	Total	42	30.9	23.4	39.46
**IL9**	Mild	1	2.9	0.15	17.05
	Moderate	1	3.8	0.2	21.58
	Severe	0	0	0	24.07
	Critical	0	0	0	13.34
	Control	0	0	0	15.02
	Total	2	1.5	0.26	5.75
IP1b	Mild	34	100	87.36	100
	Moderate	26	100	83.98	100
	Severe	16	100	75.93	100
	Critical	32	100	86.66	100
	Control	28	100	84.98	100
	Total	136	100	96.58	100
IP10	Mild	34	100	87.36	100
	Moderate	26	100	83.98	100
	Severe	16	100	75.93	100
	Critical	32	100	86.66	100
	Control	28	100	84.98	100
	Total	136	100	96.58	100
LIF	Mild	27	79.4	61.59	90.66
	Moderate	17	65.4	44.36	82.06
	Severe	10	62.5	35.87	83.72
	Critical	21	65.6	46.77	80.83
	Control	4	14.3	4.68	33.56
	Total	79	58.1	49.32	66.39
MCP1	Mild	34	100	87.36	100
	Moderate	25	96.2	78.42	99.8
	Severe	16	100	75.93	100
	Critical	32	100	86.66	100
	Control	27	96.4	79.76	99.81
	Total	134	98.5	94.25	99.74
MIP1a	Mild	22	64.7	46.47	79.7
	Moderate	19	73.1	51.95	87.65
	Severe	13	81.2	53.69	95.03
	Critical	18	56.2	37.88	73.16
	Control	11	39.3	22.13	59.27
	Total	83	61	52.27	69.16
**NGFb**	Mild	3	8.8	2.31	24.81
	Moderate	0	0	0	16.02
	Severe	2	12.5	2.2	39.59
	Critical	4	12.5	4.08	29.93
	Control	0	0	0	15.02

Cytokine - Chemokine	Group	n	%	CI 95%	
				Low	High
	Total	9	6.6	3.26	12.55
PDGFBB	Mild	34	100	87.36	100
	Moderate	26	100	83.98	100
	Severe	16	100	75.93	100
	Critical	32	100	86.66	100
	Control	28	100	84.98	100
	Total	136	100	96.58	100
PIGF1	Mild	12	35.3	20.3	53.53
	Moderate	6	23.1	9.75	44.08
	Severe	10	62.5	35.87	83.72
	Critical	23	71.9	53.02	85.6
	Control	4	14.3	4.68	33.56
	Total	55	40.4	32.22	49.21
RANTES	Mild	34	100	87.36	100
	Moderate	26	100	83.98	100
	Severe	16	100	75.93	100
	Critical	32	100	86.66	100
	Control	28	100	84.98	100
	Total	136	100	96.58	100
SCF	Mild	34	100	87.36	100
	Moderate	25	96.2	78.42	99.8
	Severe	15	93.8	67.71	99.67
	Critical	32	100	86.66	100
	Control	25	89.3	70.63	97.19
	Total	131	96.3	91.19	98.64
SDF1a	Mild	34	100	87.36	100
	Moderate	24	92.3	73.4	98.66
	Severe	15	93.8	67.71	99.67
	Critical	32	100	86.66	100
	Control	28	100	84.98	100
	Total	133	97.8	93.19	99.43
TNFa	Mild	26	76.5	58.43	88.62
	Moderate	17	65.4	44.36	82.06
	Severe	15	93.8	67.71	99.67
	Critical	23	71.9	53.02	85.6
	Control	3	10.7	2.81	29.37
	Total	84	61.8	53.01	69.85
**TNFb**	Mild	1	2.9	0.15	17.05
	Moderate	0	0	0	16.02
	Severe	0	0	0	24.07
	Critical	0	0	0	13.34
	Control	0	0	0	15.02
	Total	1	0.7	0.04	4.64
VEGFA	Mild	34	100	87.36	100

**Cytokine - Chemokine**	Group	n	%	CI 95%	
				Low	High
	Moderate	26	100	83.98	100
	Severe	16	100	75.93	100
	Critical	32	100	86.66	100
	Control	28	100	84.98	100
	Total	136	100	96.58	100
VEGFD	Mild	34	100	87.36	100
	Moderate	26	100	83.98	100
	Severe	16	100	75.93	100
	Critical	31	96.9	82	99.84
	Control	24	85.7	66.44	95.32
	Total	131	96.3	91.19	98.64

Table S2: Comparison between the value of cytokines according to their degree of severity.

Cytokine - Chemokine	Mild (N=34)		Moderate (N=26)		Severe (N=16)		Critical (N=32)	
	Med.	IQR	Med.	IQR	Med.	IQR	Med.	IQR
BDNF	60.52	184.11	58.95	205.2	53.4	28.28	66.6	111.61
EGF	1.96	8.47	2.69	11.96	1.54	13.52	2.26	2.75
Eotaxin	16.6	9.74	14.12	10.76	12.05	5.88	13.57	8.47
GMCSF	10.95	34.74	14.57	26.96	10.32	18.88	11.11	13.7
GRO-α	3.48	5.26	3.54	2.24	2.25	7.76	2.82	2.61
HGF	128.75	123.09	137.48	89.1	155.25	210	411.25	602.96
IFN-α	0.75	2.76	0.59	1.55	0.32	2.42	0.42	0.46
IFN-γ	7.88	5.74	9.36	6.17	7.04	12.67	9.72	8.28
IL-1α	1.33	6.32	1.51	6.74	3.69	7.19	3.14	11.54
IL-1β	6.88	11.02	6.28	7.01	6.55	10.53	6.61	10.72
IL-10	1.62	1.54	1.8	1.13	1.68	3.19	1.8	2.88
IL-13	2.37	3.38	2.51	2.97	1.2	1.67	2.25	1.71
IL-15	13.3	19.51	13.94	15.56	13.82	10.82	8.33	21.65
IL-17α	8.03	12.53	5.9	7.96	7.2	15.55	6.35	19.81
IL-18	46.38	48.1	39.17	42.05	27.73	58.84	60.92	61.38
IL1-RA	488	731.72	652.6	741.21	556	1200.88	718.67	1332
IL-2	15.02	21.64	12.52	19.7	18.38	16.77	10.91	17.57
IL-22	1.73	13.21	6.96	22.03	1.23	16.41	9.43	56
IL-27	19.5	48.36	24.82	35.34	7.41	12.87	20.44	22.58
IL-4	4.92	7.07	7.88	6.47	4.55	6.24	5.82	4.91
IL-5	4.25	19.5	9.94	15.02	3.32	4.85	6.91	45.94
IL-6	10.61	16.33	16.23	20.28	20.27	34.54	8.18	27.14
IL-7	1.69	2.93	1.45	2.73	1.88	2.71	1.57	3.06
IL8	1.79	2.41	2	6.93	1.87	5.51	2.08	5.06
IP-1β	45	39.61	39.29	49.35	51.12	41.62	56.7	39.52
IP-10	43.83	41.91	49	29.8	35.67	45.23	46.78	41.15
LIF	17.32	15.81	13.53	10.07	11.81	17.75	13.48	20.18

MCP1	42.83	27.01	32.77	20.51	36.97	28.25	54.28	63.05
MIP-1α	3.37	12.1	3.34	7.04	3.98	14.34	2.51	4.7
PDGFBB	189.25	508.73	241.75	563.48	212	243	639.5	1207
PIGF1	2.86	34.82	4.15	9.03	39.42	169.11	29.15	102.98
RANTES	21.35	20.81	22.04	10.74	24.92	25.99	22.95	12.92
SCF	5.94	5.32	7.86	5.72	6.84	4.4	7.1	8.71
SDF-1α	632.5	396.5	610.5	471.38	651.5	971	736.75	832.92
TNF-α	5.61	11.59	5.87	7.3	6.29	9.28	7.92	10.39
VEGFA	96.72	144.68	107.55	90.48	153	152.52	216.83	282.12
VEGFD	13.02	11.48	12.9	13.86	13.72	12.2	11.3	9.59

Med, Median; IQR, interquartile range.

Table S3: Likelihood-ratio test (LRT) to check the assumption of proportional odds by comparing the proportional odds model with a multinomial model.

Cytokine	Log Likelihood		LRT	p-value
	M1	M2		
BDNF	-145.394	-140.8061	9.1757	***0.0102***
EGF	-145.2106	-141.2928	7.8357	***0.0199***
Eotaxin	-144.8607	-140.3148	9.0918	***0.0106***
GMCSF	-145.2246	-141.0904	8.2684	***0.016***
GROa	-144.9565	-140.7072	8.4986	***0.0143***
HGF	-129.1737	-122.0511	14.2452	***0.0008***
IFNa	-144.782	-140.8383	7.8875	***0.0194***
IFNg	-145.2699	-140.5105	9.5188	***0.0086***
IL1a	-143.556	-139.3312	8.4497	***0.0146***
IL1b	-144.7608	-140.877	7.7676	***0.0206***
IL10	-145.2825	-141.6318	7.3014	***0.0260***
IL13	-145.2908	-140.2268	10.1281	***0.0063***
IL15	-143.1028	-138.9887	8.2282	***0.0163***
IL17a	-145.0937	-140.9091	8.3692	***0.0152***
IL18	-144.856	-138.6082	12.4957	***0.0019***
IL1RA	-145.333	-141.7225	7.221	***0.0270***
IL2	-144.2746	-139.8474	8.8544	***0.0119***
IL22	-144.9003	-140.172	9.4566	***0.0088***
IL27	-145.2467	-139.8769	10.7395	***0.0047***
IL4	-145.4409	-140.766	9.3498	***0.0093***
IL5	-145.428	-139.3138	12.2284	***0.0022***
IL6	-145.4587	-140.0344	10.8487	***0.0044***
IL7	-145.4341	-141.7656	7.3371	***0.0255***
IL8	-145.2873	-141.1431	8.2884	***0.0159***
IP1b	-145.2169	-141.375	7.6837	***0.0215***
IP10	-145.396	-140.8723	9.0474	***0.0108***
LIF	-145.4514	-140.6877	9.5273	***0.0085***
MCP1	-143.782	-136.9834	13.5972	***0.0011***
MIP1a	-145.458	-141.5284	7.8591	***0.0197***
PDGFBB	-142.6643	-136.623	12.0826	***0.0024***
PIGF1	-142.0374	-137.6666	8.7416	***0.0126***
RANTES	-145.2314	-140.8309	8.8009	***0.0123***
SCF	-145.1679	-141.3803	7.5753	***0.0226***
SDF1a	-145.0041	-140.804	8.4002	***0.0150***
TNFa	-145.3963	-141.73	7.3326	***0.0256***
VEGFA	-143.154	-139.2963	7.7154	***0.0211***
VEGFD	-144.9091	-141.1052	7.6079	***0.0223***
